# Effect of acute exposure to nonylphenol on biochemical, hormonal, and hematological parameters and muscle tissues residues of Nile tilapia; *Oreochromis niloticus*

**DOI:** 10.14202/vetworld.2016.616-625

**Published:** 2016-06-20

**Authors:** Hager Tarek H. Ismail, Heba Hassan H. Mahboub

**Affiliations:** 1 Department of Clinical Pathology, Faculty of Veterinary Medicine, Zagazig University, Zagazig City, Sharkia Province, Egypt; 2 Department of Fish Diseases and Management, Faculty of Veterinary Medicine, Zagazig University, Zagazig City, Sharkia Province, Egypt

**Keywords:** biochemical, hematological, hormonal, Nile tilapia, nonylphenol

## Abstract

**Aim:**

This study was aimed to evaluate some biochemical, hormonal, hematological, and histopathological changes in Nile tilapia, *Oreochromis niloticus*, after acute exposure to nonylphenol (NP). In addition to detection of NP residues in the fish, muscle tissues for human health concern.

**Materials and Methods:**

A total of 90 apparently healthy Nile tilapia, *O. niloticus*, were randomly divided into three equal groups; each containing 30 fish (three replicates). Groups 1 and 2 kept as a control and solvent control (acetone), respectively, and Group 3 exposed to NP at a dose level of 500 µg/L water for 7 successive days. Blood and tissue samples were collected 2 times randomly from each group after 7 days from fish exposure to NP and 10 days from exposure stopping.

**Results:**

Fish exposed to NP Group 3 showed anorexia, sluggish movement, erythema of the skin, areas of scales loss, and hemorrhagic ulcers in some areas of body region leading to exposing the viscera. Biochemical results revealed a significant increase in serum total proteins and globulins levels, a highly significant increase in serum alanine aminotransferase and aspartate aminotransferase activities, triglycerides, cholesterol, and creatinine levels, insignificant increase in serum uric acid level, and a highly significant decrease in serum testosterone and estradiol-β17 levels in Group 3 in compare with the control group. Histopathological finding confirms these results. While hematological results of the same group revealed a significant increase in red blood cells count and packed cell volume value, insignificant increase in hemoglobin concentration, leukopenia, lymphopenia, and monocytopenia in compared with the control group. All of these changes appeared after 7 days from fish exposure to NP. Most of these alterations returned toward the normal level after 10 days from stopping exposure to NP. NP residues detected in fish muscle tissues of Group 3 during exposure and after stopping exposure to it.

**Conclusion:**

It is concluded that NP is a toxic pollutant and has an adverse effect on fish health and reproduction as well as accumulates in fish muscle tissues which may cause human health hazard.

## Introduction

Nile tilapia, *Oreochromis niloticus*, is a worldwide fish species in aquaculture because of its adaptive capability to different environmental conditions and types of diets, meat palatability, high nutritional value and ability to reproduce in captivity with low expense [1].

Water pollution is a discharging of any material into water that causes acute or short-term and sometimes chronic or long-term damage to the ecosystem, which considers one of the most concerned issues nowadays. A countless number of chemical compounds that through into aquatic ecosystems without any pre-treatment can cause a dangerous impact on marine and freshwater organisms and human [2-4].

Nonylphenol (NP) originates as a product of NP ethoxylates decomposition. It is a non-ionic surfactant, which discharged into the aquatic environment and used worldwide in the formation of detergents, paints, lubricants, polystyrene tubes, insecticides, herbicides, paper, textile, and many other industries [5,6]. It has been detected in samples of surface water, rivers, sewage sludge and effluents, sediments, and estuaries and revealing a wide range of NP concentrations reaching even higher than 100 µg/L up to 644 µg/L water [7].

NP has toxic, weak estrogenic, and carcinogenic effects in fish, birds, and mammals beside its resistance toward biodegradation [8,9]. It was detected to be toxic (lethal concentration 50) to fish at concentrations from 17 to 3000 µg/L water [10]. Relatively, low concentrations of NP can lead to fish death [7].

Measurement serum biochemical parameters can be useful to identify target organs of toxicity as well as early warning of potentially damaging changes and general health status of living organisms under stress [11]. Changes in the hormones concentration, especially those regulating vital functions such as reproduction may consider as early warning indicators of toxicity stress in fish. Circulating hormones levels give indication about the sublethal effects of many chemicals [12]. Furthermore, different hematological parameters help in evaluating the response of different types of blood cells in the condition of stress due to toxicity and quickly reflect the poor condition of fish [13].

However, NP concentration in the fish muscles was moderately low, but the muscles, consider only tissue where active form was found, not conjugated with other compounds, which indicating that long exposure time will lead to a continuous NP accumulation in the muscles, which represent the majority of the fish’s body mass [14]. Hence, increasing NP adverse effects due to their strong ability to bio-accumulate in fish which follow by biomagnifications through the food chain in the higher living organisms [15].

The aim of this study was to evaluate the impact of acute exposure to NP on some biochemical, hormonal, and hematological parameters as well as histopathological alterations of liver, kidneys, and testes in Nile tilapia, *O. niloticus*. Beside evaluation of the residues levels of NP in the fish muscle tissues for human health concern.

## Materials and Methods

### Ethical approval

All procedures of the current experiment were carried out in accordance with the Egyptian laws and university guidelines for the care of experimental animals and have been approved by the Committee of the Faculty of Veterinary Medicine, Zagazig University, Egypt.

### Experimental fish

A total of 90 apparently healthy, adult Nile tilapia, *O. niloticus*, were obtained alive from Abbassa private fish farms, Sharkia Province, Egypt, with an average body weight of 100±10 g. The fish were randomly stocked in nine glass aquaria (each 80 cm × 60 cm × 30 cm) at a rate of 10 fish per 80-L of dechlorinated tap water and supplied by aerators at temperature 24±2°C, pH 7±0.2, and dissolved oxygen 5-6 mg/L. Fish were fed twice daily with a balanced commercial fish diet in a rate of 3% of fish body weight during the experimental period. Fish were acclimated for a period of 2-weeks in the laboratory conditions before the experiment.

### Chemical

NP, technical grade, mixture of ring, and chain isomers were purchased from Sigma-Aldrich (Sigma-Aldrich Chemical Co., St. Louis, MO, USA). A stock solution was prepared by dissolving NP in acetone and storing in the dark at 4°C. The volume of acetone was kept equal in solvent control and NP-treated groups.

### Experimental design

Fish were randomly divided into three equal groups: Each group has three replicate (10 fish per replicate). Groups 1 and 2 kept as a control and solvent control (acetone), respectively, and Group 3 exposed to NP at a dose level of 500 µg/L water [16]. Duration of fish exposure to NP was 7 successive days, then stopping fish exposure to the chemical for 10 successive days. Water and NP were completely replenished each 24 h to maintain the chemical. Clinical signs and lesions were observed as well as mortality rate was recorded during the experimental period.

### Sampling

Samples were collected randomly from fish in each group after 7 days from exposure to NP and 10 days from stopping exposure. Blood samples were collected from fish caudal vein and were divided into two portions. The first portion was collected into the plain centrifuge tube without anticoagulant for serum separation for biochemical analysis. The second portion was collected into clean Wasserman tubes containing dipotassium salts of ethylenediamine tetraacetic acid for hematological analysis. Three surviving fish from each group were removed and sacrificed for collecting samples from liver, kidneys, and testes for histopathological examination and muscle tissues for determination of NP residues.

### Biochemical studies

Serum was used to determine total proteins and albumin levels according to Burtis and Ashwood [17], globulins level according to Doumas *et al*. [18], alanine aminotransferase (ALT) and aspartate aminotransferase (AST) activities according to Burtis and Ashwood [17], triglycerides level according to Kaplan *et al*. [19], cholesterol level according to Meiattini [20], creatinine level according to Burtis and Ashwood [17], and uric acid level according to Tietz [21]. All of these parameters were measured using specific reagent kits purchased from Diamond Diagnostic Company and Spinreact. Serum testosterone and estradiol-β17 (E2) levels were determined according to Wheeler [22] and Melmed *et al*. [23], respectively. They were measured using reagent kits provided by Roche Diagnostics International Ltd.

### Hematological studies

Total erythrocytic and leukocytic counts, packed cell volume (PCV) value, and hemoglobin (Hb) concentration were determined using automated blood cell analyzer (Sysmex XT-2000iV, Kobe, Japan) [24]. Giemsa-stained blood films were done for estimation of differential leukocytic count and detection of nuclear abnormalities of erythrocytes, respectively [25,26].

### Histopathological studies

Liver, kidneys, and testes of fish were dissected out and then fixed in 10% neutral buffered formalin, dehydrated in a graded ethanol series, cleared in xylene, and finally embedded in paraffin wax. Paraffin sections of 5 µ thickness were stained by hematoxylin and eosin and examined microscopically [27].

### Residual analysis

Muscle tissues were sampled, frozen then homogenized in methanol and centrifuged at 3000 rpm for 15 min. Supernatant was collected and dried under nitrogen gas. The samples were reconstituted in 100 ml methanol [28] and subjected to separation by high-performance liquid chromatography (HPLC) with the following condition: Flow rate 1/min, Agilent 1100 series (Waldborn, Germany), quaternary pump (G1311A), degasser (G1322A), thermostated auto samples (G1329A), variable wavelength detector (G1314A), and column Zorbax 300SB C18 column (Agilent Technologies, USA). Injection was carried out at wavelength 280 nm for separation. The mobile phase was composed of Solvent A - water and Solvent B - acetonitrile. The run consisted of a 40 min linear gradient from 50% A to 50% B in 3 min to 34% A and 66% B in 17 min, to a final solvent ratio for next 20 min. Flow rate was 1 ml/min. NP was eluted at 23.65±0.8 min. Laboratory reagents were of analytical and HPLC grade purchased from Sigma. Data were expressed as µg/g tissue weight [29].

### Statistical analysis

Data obtained from this investigation were analyzed statistically using F-test [30]. Means in the same raw followed by different letters were significantly different, and the highest value was represented by the letter (a).

## Results and Discussion

Environmental toxicants pollution has become one of the most critical problems all over the world. Fish are specially exposed to these pollutants because the pollution end up in the aquatic environment regardless of where it occurs [31]. NP is a ubiquitous pollutant which has damaging effects on important physiological functions of fish through induction of apoptosis and oxidative damage of different organs [32].

**Figure-1:**
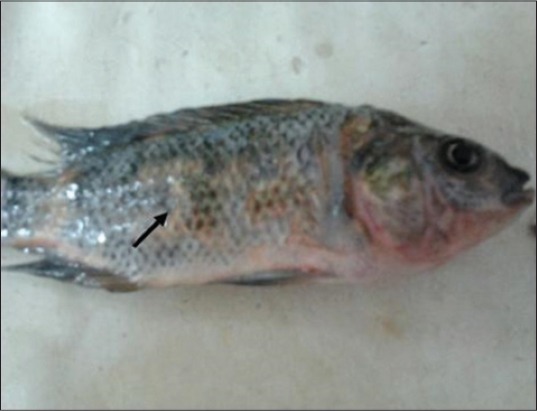
Nile tilapia, *Oreochromis Niloticus*, in Group 3 showing ulceration and erythema of the skin beside scales loss at 4^th^ day from exposure to nonylphenol.

**Figure-2:**
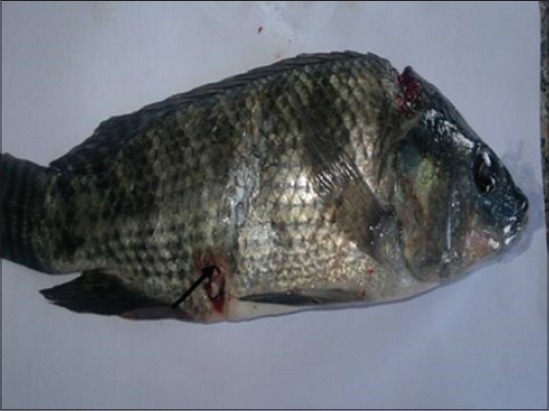
Nile tilapia, *Oreochromis niloticus*, in Group 3 showing areas of scales loss and hemorrhagic ulcers in some areas of body region leading to exposing the viscera at 6^th^ day from exposure to nonylphenol.

**Figure-3:**
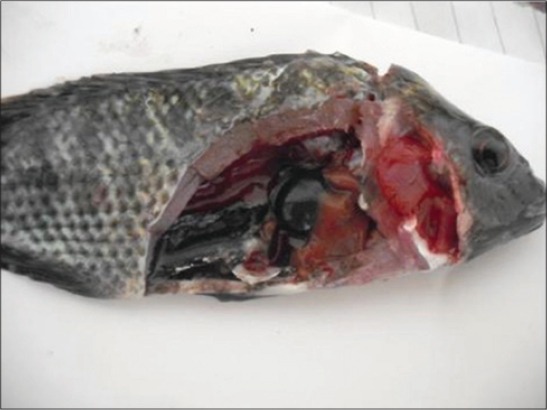
Post-mortem findings of Nile tilapia, *Oreochromis Niloticus*, in Group 3 showing severe congestion in gills, liver, spleen, and kidneys beside enlargement of gall bladder after 7 days from fish exposure to nonylphenol.

Concerning the clinical signs, control and solvent control groups appeared healthy along experimental period, whereas the majority of fish exposed to NP showed sluggish movement, anorexia, aggregate near the aquarium side, loss of escape reflex, and gasping air from the surface at 2^nd^ day of exposure to NP, ulceration, and erythema of the skin beside scales loss were observed at 4^th^ day of exposure to NP (Figure-1), while at 6^th^ day after exposure to NP, areas of scales loss and hemorrhagic ulcers in some areas of body region leading to exposing the viscera were detected (Figure-2). The post-mortem lesions were severe congestion in gills, liver, spleen, and kidneys beside enlargement of the gallbladder (Figure-3).

No fish mortality recorded in control and solvent control groups during the experimental period while the mortality rate reached 40% after 7 days from fish exposure to NP and 13.34% after 10 days from stopping exposure to NP. All clinical signs appeared on fish in Group 3 beside mortality rate related to toxic effects of NP on vital organs and its functions.

Serum total proteins are often used as an index of the physiological status of the fish, as they are considered one of the most stable components of blood, which impacted by a few factors [33]. In the presented study, results showed a significant increase in serum total proteins and globulins levels in Group 3 after 7 days from fish exposure to NP in compare with control group may be due to immune response toward the toxicity stress raises the globulins level [34], and therefore, the total proteins level is increased (Table-1). After 10 days from stopping fish exposure to NP, serum total proteins and globulins levels (Table-2) returned toward the normal value, whereas serum albumin level showed insignificant increase may be due to stabilizing fish condition after stopping chemical pollutant stress.

**Table-1:** Some serum biochemical parameters of Nile tilapia, *O. niloticus* (mean values±SE) after 7 days from fish exposure to NP.

Parameters	Groups			F test

	Group 1 control	Group 2 solvent control (acetone)	Group 3 NP	
Total proteins (g/dl)	2.23^b^±0.16	2.57^ab^±0.12	3.07^a^±0.20	*
Albumin (g/dl)	1.00±0.12	0.91±0.05	1.00±0.06	NS
Globulins (g/dl)	1.28^b^±0.10	1.65^ab^±0.15	2.01^a^±0.17	*
ALT (U/L)	2.94^b^±0.41	2.56^b^±0.13	18.94^a^±2.21	**
AST (U/L)	19.27^b^±2.00	72.76^a^±7.68	86.58^a^±3.36	**
TG (mg/dl)	92.53^b^±8.50	64.78^b^±13.94	146.27^a^±5.47	**
Cholesterol (mg/dl)	136.53^b^±9.03	151.68^b^±11.53	217.53^a^±3.44	**
Creatinine (mg/dl)	0.99^b^±0.02	0.64^b^±0.04	1.55^a^±0.21	**
Uric acid (mg/dl)	1.00^ab^±0.01	0.70^b^±0.03	1.29^a^±0.26	*

Means in the same row with different superscript letters are significantly different.

** Highly significant difference at p≤0.01,

* Significant difference at p≤0.05.

NS=Non significant, SE=Standard error, ALT=Alanine aminotransferase, AST=Aspartate aminotransferase, TG=Triglycerides, NP=Nonylphenol, *O. niloticus*=*Oreochromis niloticus*

**Figure-4:**
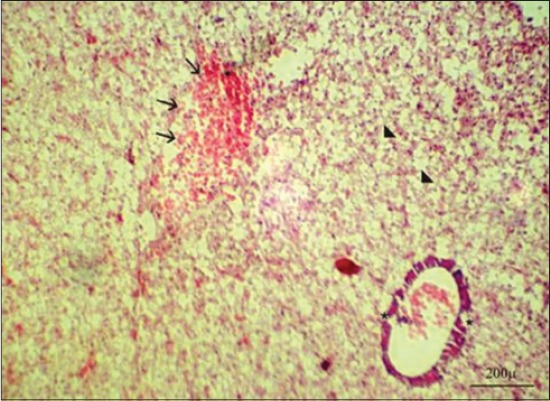
Liver of Nile tilapia, *Oreochromis Niloticus*, in Group 3 showing sever degenerative changes in the hepatic tissues represented by vacuolation of the hepatic cells (arrowheads), telangiectasia (arrows), and hepatopancreatic necrosis (asterisks) after 7 days from fish exposure to nonylphenol, H and E, Bar: 200 µm.

**Figure-5:**
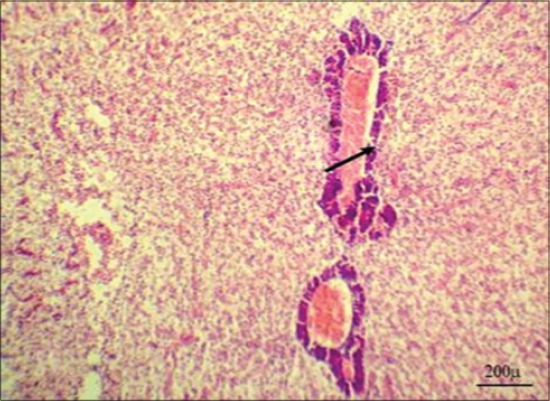
Liver of Nile tilapia, *Oreochromis niloticus*, in Group 3 showing normal hepatic architecture with mild congestion in the hepatic blood vessels and slight vacuolation of the hepatic cells after 10 days from stopping fish exposure to nonylphenol, H and E, Bar: 200 µm.

Serum enzymes such as ALT and AST could be utilized as a sensitive marker for toxicity, which gave an early warning of hazardous alterations in polluted aquatic living organisms [31]. In the present study, results indicated a highly significant increase in ALT and AST activities in Group 3 after 7 days from fish exposure to NP in compare with control group may be due to hepatic damage and liberation of large quantities of these enzymes into the blood stream as a liver is a rich organ with those enzymes (Table-1) [35]. Our results were confirmed by histopathological findings of liver, which showing severe degenerative changes in the hepatic tissues represented by vacuolation of the hepatic cells, telangiectasia, and hepatopancreatic necrosis (Figure-4). Those enzymes showed a significant increase in the same group (Table-2) but with a lesser degree after 10 days from stopping fish exposure to NP in compare with control group may be due to lowering NP toxicity impact and improvement liver condition. Our results were confirmed by histopathological findings of liver, which showing normal hepatic architecture with mild congestion in the hepatic blood vessels and slight vacuolation of the hepatic cells (Figure-5). Highly significant and significant increase in serum AST in Group 2 along experimental period in compare with control group could be due to slight toxicity effects of acetone on different body organs.

**Table-2:** Some serum biochemical parameters of Nile tilapia, *O. niloticu*s (mean values±SE) after 10 days from stopping fish exposure to NP.

Parameters	Groups			F test

	Group 1 control	Group 2 solvent control (acetone)	Group 3 NP	
Total proteins (g/dl)	2.71±0.27	2.10±0.20	2.80±0.29	NS
Albumin (g/dl)	1.08^ab^±0.07	0.83^b^±0.10	1.25^a^±0.07	*
Globulins (g/dl)	1.62±0.22	1.27±0.11	1.55±0.23	NS
ALT (U/L)	2.98^b^±0.42	2.88^b^±0.29	5.35^a^±1.00	*
AST (U/L)	18.51^b^±1.97	36.04^a^±3.69	43.68^a^±8.27	*
TG (mg/dl)	64.89^b^±9.81	78.87^b^±10.50	229.57^a^±16.04	**
Cholesterol (mg/dl)	158.53^b^±13.84	165.47^b^±6.85	209.01^a^±5.76	**
Creatinine (mg/dl)	0.92±0.20	0.79±0.03	0.83±0.14	NS
Uric acid (mg/dl)	0.94^b^±0.05	0.62^b^±0.03	1.48^a^±0.17	**

Means in the same row with different superscript letters are significantly different.

** Highly significant difference at p≤0.01,

* Significant difference at p≤0.05.

NS=Non significant, SE=Standard error, ALT=Alanine aminotransferase, AST=Aspartate aminotransferase, TG=Triglycerides, SE=Standard error, *O. niloticus*=*Oreochromis niloticus,* NP=Nonylphenol

Serum triglycerides and cholesterol levels (Tables-1 and 2) showed a highly significant increase in Group 3 along experimental period in compare with control group could be due to mobilize triglycerides under chemical stress to meet an increased request for energy to overcome damaging conditions occurred by the toxicant/xenobiotic and to meet energy required to support increased physical activity, bio-transformation, and discharge of xenobiotic [36]. Liver dysfunction and inhibition of enzymes which convert cholesterol into the bile acid lead to observed hypercholesterolemia [37]. Increasing of those parameters continues in Group 3 after 10 days from stopping fish exposure to NP in compare with control group indicated fish physiological trials to overcome toxicant effects.

**Figure-6:**
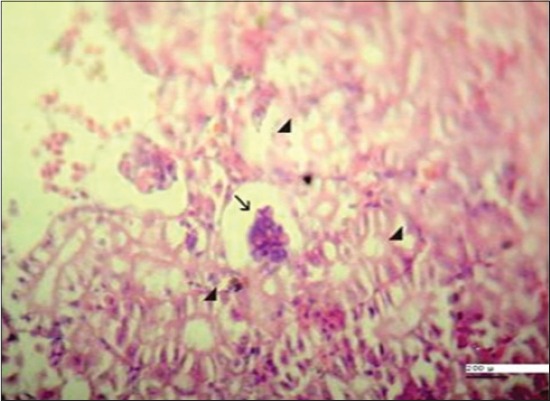
Kidney of Nile tilapia, *Oreochromis niloticus*, in Group 3 showing marked vaculation in the epithelium of the renal tubules (arrowheads) with appearance of shrunken glomeruli (arrow) after 7 days from fish exposure to nonylphenol, H and E, Bar: 200 µm.

**Figure-7:**
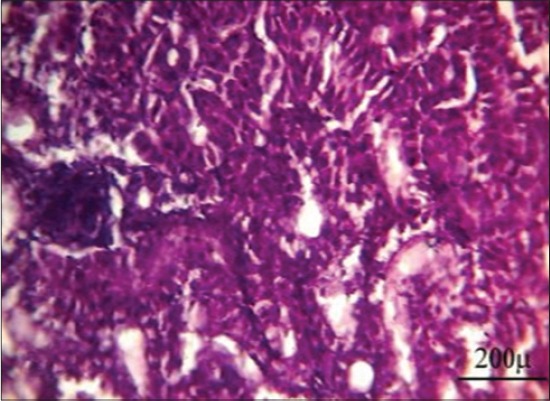
Kidney of Nile tilapia, *Oreochromis niloticus*, in Group 3 showing normal histological architecture of the renal tissues after 10 days from stopping fish exposure to nonylphenol, H and E, Bar: 200 µm.

Serum creatinine and uric acid levels can be used as a rough index of the glomerular filtration rate and markers of impairment in kidney functions [38]. In our study, results showed highly significant and insignificant increase in serum creatinine and uric acid values, respectively, in Group 3 after 7 days from fish exposure to NP in compare with control group may be due to kidney dysfunction, which lead to reduce renal blood flow with reduction in glomerular filtration rate and decrease in creatinine and uric acid excretion resulting in azotemia [39] (Table-1). Our results were confirmed by histopathological findings of kidney which showing marked vacuolation in the epithelium of the renal tubules with appearance of shrunken glomeruli (Figure-6). Non-significant changes in serum creatinine level (Table-2) appeared in the same group after 10 days from stopping fish exposure to NP which indicated improvement renal function after removal pollutant stress, while serum uric acid level showed highly significant increase in the same group in compare with control group may be due to increased muscular tissue catabolism, increased synthesis or decreased degradation of these compounds [40]. Our results were confirmed by histopathological findings of kidney which showing normal histological architecture of the renal tissues (Figure-7).

**Figure-8:**
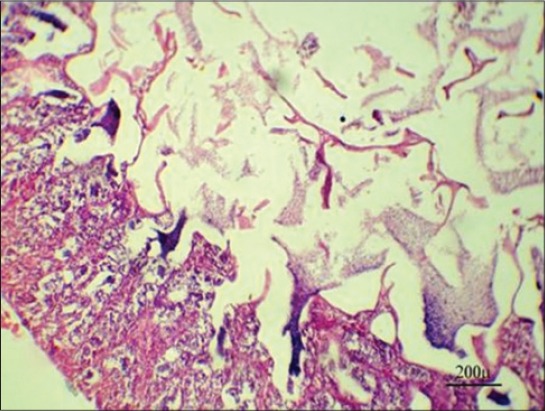
Testis of Nile tilapia, *Oreochromis niloticus*, in Group 3 showing minimal spermatogenesis activity with the presence of few spermatozoa after 7 days from fish exposure to nonylphenol, H and E, Bar: 200 µm.

**Figure-9:**
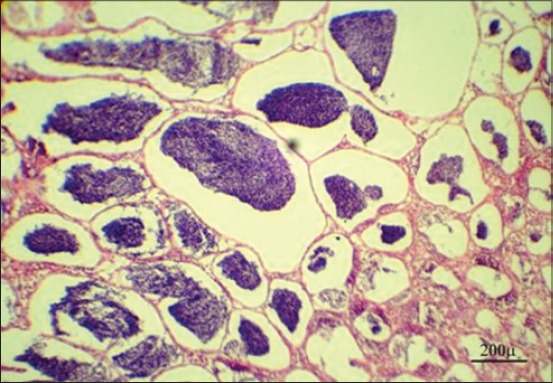
Testis of Nile tilapia, *Oreochromis niloticus*, in Group 3 showing marked improvement in the spermatogenesis activity with presence of huge number of spermatozoa in the seminiferous tubules after 10 days from stopping fish exposure to nonylphenol, H and E, Bar: 200 µm.

Steroid hormones are one of the several hormones that influence fish reproduction. The major androgens produced by testicular tissue differ from the fish species to another beside developmental stages and include testosterone, 11-ketotestosterone, and androstenedione [41]. In our study, results showed a highly significant decrease in serum testosterone level in Group 3 after 7 days from fish exposure to NP in compare with control group may be due to NP act by indirect way via the hypothalamus-pituitary axis to change gonadotropin synthesis and secretion which lead to interrupt of sex steroid production, which have secondary effects on the normal function of the testicular cells or it acts directly on the testicular cell either by general cytotoxic effect which leads to damage of the testis cells, or endocrine, in which the function of specific cells (e.g., Sertoli cells) are disrupted due to an endocrine malfunction [42] (Table-3). Our results were confirmed by histopathological findings of testis which showing minimal spermatogenesis activity with the presence of few spermatozoa (Figure-8). After 10 days from stopping fish exposure to NP, testosterone hormone level returns toward the normal value in Group 3, which indicates reversing NP cytotoxic and hormonal disturbing effect toward normal physiological function (Table-4). Our results were confirmed by histopathological findings of the testis, which showing marked improvement in the spermatogenesis activity with the presence of huge numbers of spermatozoa in the seminiferous tubules (Figure-9).

**Table-3:** Serum testosterone and estradiol-β17 (E2) levels of Nile tilapia, *O. niloticus* (mean values±SE) after 7 days from fish exposure to NP.

Parameters	Groups			F test

	Group 1 control	Group 2 solvent control (acetone)	Group 3 NP	
Testosterone (ng/ml)	1.54^a^±0.08	2.46^a^±0.74	0.12^b^±0.002	**
Estradiol-β17 (E2) (pg/ml)	578.60^a^±167.60	330.15^a^±23.22	22.08^b^±1.99	**

Means in the same row with different superscript letters are significantly different.

** Highly significant difference at p≤0.01.

SE=Standard error, *O. niloticus*=*Oreochromis niloticus*, NP=Nonylphenol

**Table-4:** Serum testosterone and Estradiol-β17 (E2) levels of Nile tilapia, *O. niloticus* (mean values±SE) after 10 days from stopping fish exposure to NP.

Parameters	Groups			F test

	Group 1 control	Group 2 solvent control (acetone)	Group 3 NP	
Testosterone (ng/ml)	1.52±0.09	2.44±0.74	2.17±0.11	NS
Estradiol-β17 (E2) (pg/ml)	589.70±166.40	357.03±9.41	307.05±47.25	NS

NS=Non significant, SE=Standard error, *O. niloticus*=*Oreochromis niloticus*, NP=Nonylphenol

Estradiol-β17 (E2) is an important steroid hormone for the coordination of different responses (developmental, physiological, and behavioral), which are fundamental for the fish reproduction [43]. In the present study, results indicated a highly significant decrease in 17-estradiol (E2) hormone level in Group 3 after 7 days from fish exposure to NP in compare with control group may be due to increase steroid metabolizing enzymes activities which lead to increase in hormone clearance (Table-3). In addition, NP may have a direct effect on the E2 feedback system or gonadotropin synthesis in the pituitary gland [44,45]. After 10 days from stopping fish exposure to NP, estradiol-β17 hormone level returns toward the normal value which indicates lowering hormonal disturbing effect of NP (Table-4).

Measurement of hematological parameters is an important tool to detect nutritional, physiological, and pathological changes in fish [46].

Regarding the erythrogram results, Table-5 revealed a significant increase in red blood cells count and PCV value and insignificant increase in Hb concentration in Group 3 after 7 days from fish exposure to NP in compare with control group may be due to compensatory erythropoiesis which occurs by stimulation of erythropoietin hormone due to elevated demands for O_2_ or CO_2_ transportation as a result of destruction of gill membranes which a common consequence of exposure to NP causing faulty gaseous exchange with asphyxiation [47]. Those parameters returned to the normal level (Table-6) in Group 3 after 10 days from stopping fish exposure to NP in compare with control group indicate that changes in erythrogram are temporary and revisable depend on decrease toxicity effect of NP on vital organs as gills.

**Table-5:** Hemogram parameters of Nile tilapia, *O. niloticus* (mean values±SE) after 7 days from fish exposure to NP.

Parameters	Groups			F test

	Group 1 control	Group 2 solvent control (acetone)	Group 3 NP	
RBCs (×10^6^⁄µl)	1.13^b^±0.9	1.33^ab^±0.04	1.48^a^±0.11	*
PVC (%)	18.20^b^±1.05	22.34^ab^±1.26	24.02^a^±1.99	*
Hb (g %)	4.70^ab^±0.44	4.61^b^±0.18	5.76^a^±0.36	*
WBCs (×10^3^µl)	28.58^a^±0.40	25.19^ab^±3.15	20.37^b^±0.96	*
Lymphocytes (×10^3^/µl)	17.49^a^±1.50	15.54^ab^±2.05	12.43^b^±0.22	*
Heterophils (×10^3^/µl)	8.78±1.01	8.65±1.98	7.58±0.66	NS
Eosinophils (×10^3^/µl)	0.38±0.24	0.69±0.28	0.18±0.18	NS
Monocytes (×10^3^/µl)	1.93^a^±0.33	0.31^b^±0.20	0.18^b^±0.18	**

Means in the same row with different superscript letters are significantly different.

** Highly significant difference at p≤0.01,

* Significant difference at p≤0.05,

NS=Non significant, SE=Standard error, RBCs=Red blood corpuscles, PCV=Packed cell volume, Hb=Hemoglobin, WBCs=White blood corpuscles, SE=Standard error, *O. niloticus*=*Oreochromis niloticus,* NP=Nonylphenol

**Table-6:** Hemogram parameters of Nile tilapia, *O. niloticus* (mean values±SE) after 10 days from stopping fish exposure to NP.

Parameters	Groups			F test

	Group 1 control	Group 2 solvent control (acetone)	Group 3 NP	
RBCs (×10^6^⁄µl)	1.31±0.03	1.36±0.18	1.50±0.12	NS
PVC (%)	20.92±1.32	19.34±2.76	26.00±3.02	NS
Hb (g %)	4.82±0.37	4.06±0.49	5.44±0.69	NS
WBCs (×10^3^/µl)	27.00^b^±1.78	23.00^b^±1.95	36.30^a^±3.81	*
Lymphocytes (×10^3^/µl)	16.09^ab^±2.07	13.34^b^±2.08	22.27^a^±1.87	*
Heterophils (×10^3^/µl)	8.90^ab^±1.00	7.45^b^±0.43	12.63^a^±1.83	*
Eosinophils (×10^3^/µl)	0.38±0.24	1.00±0.10	0.35±0.35	NS
Monocytes (×10^3^/µl)	1.63±0.37	1.21±0.60	1.05±0.43	NS

Means in the same row with different superscript letters are significantly different,

* Significant difference at p≤0.05.

NS=Non significant, SE=Standard error, RBCs=Red blood corpuscles, PCV=Packed cell volume, Hb=Hemoglobin, WBCs=White blood corpuscles, *O. niloticus*=*Oreochromis niloticus*, NP=Nonylphenol

**Figure-10:**
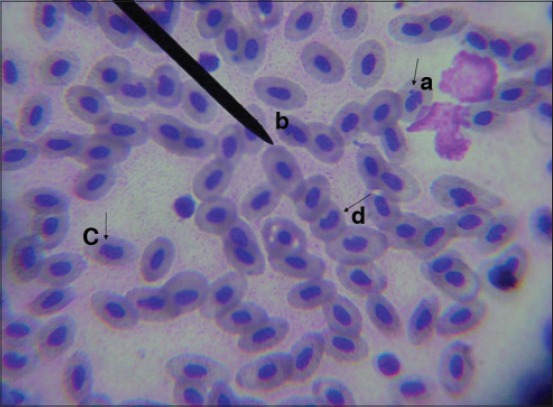
Nuclear abnormalities in peripheral blood erythrocytes of Nile Tilapia, *Oreochromis niloticus*, in Group 3 after 7 days from fish exposure to nonylphenol showing of micronucleus (a), binucleated nucleus (b), blebbed nucleus (c), and kidney-shaped nucleus (d).

Several erythrocytes morphological abnormalities are effective indicators of cytotoxicity and a process by which the cell eliminates any amplified genetic material from the nucleus lead to formation of nuclear abnormalities [48]. In our study, genotoxic alterations include formation of micronucleus (Figure-10a), binucleated nucleus (Figure-10b), blebbed nucleus (Figure-10c), and kidney-shaped nucleus (Figure-10d) appeared in Group 3 after 7 days from fish exposure to NP. Micronuclei are masses of chromatin appearing as small nuclei outside the nucleus which originate from either the breakage of chromosomes, which leads to the formation of chromosome fragments or dysfunction of the mitotic spindle apparatus that leads to entire chromosomes lagging behind in the anaphase stage and fail to become incorporated into daughter cell nuclei during cell division [49]. The binuclei and blebbed nucleated cells have the same origin as micronuclei and are considered as genotoxic analogs of micronuclei [50]. Binuclear cell formation is a marker for abnormal cell division due to blocking of cytokinesis, while blebbed nuclei may be a precursor of micronuclei [51]. Kidney-shaped nucleus may consider different precursors of micronuclei or binuclei phenomena [52].

Regarding the leukogram results, Table-5 revealed leukopenia, lymphopenia, and monocytopenia in Group 3 after 7 days from fish exposure to NP in compare with control group may be due to the immunosuppression status of fish after acute exposure to toxic substances [53].

Leukocytosis, non-significant lymphocytosis, and heterophilia (Table-6) in Group 3 after 10 days from stopping fish exposure to NP in compare with control group may be due to an increase in immunity which helps in survival and recovery of the fish exposed to toxicants. Furthermore, leukocytes help in the removal of cellular debris of necrosed tissue at a higher rate. Leukocytosis considered to be an adaptive way for the fish tissues under chemical toxicant stress [54].

In the presented study, the NP residues in fish muscle tissue samples from three randomly selected fish ranged from 0.019 to 0.118 µg/g tissue weight after 7 days from exposure to NP, whereas ranged from 0.031 to 0.053 µg/g tissue weight after 10 days from stopping exposure to NP which suggested that NP accumulate in fish muscles during and even after stopping fish exposure to it (Table-7). Since muscle tissues form large biomass of fish so they act as a depot for storage of NP and consider as an edible food part, so low NP levels can have great impact on fish and human health. The presence of low concentrations of NP in muscle tissue samples from the control and solvent control groups may be due to uncontrolled exposure of the fish to NP either from water storage tanks (plastic tanks, water pipes, and metal/plastic taps) or water chlorination, which is a common treatment process for water can increase the formation of NP metabolites [29].

**Table-7:** NP residues in muscle tissues of Nile tilapia, *O. niloticus* after 7 days from fish exposure (S1) and 10 days from stopping fish exposure to it (S2).

Samples	NP concentration (µg/g tissue weight)
Group 1 control	0.005
Group 2 solvent control (S1)	0.021
Group 3 NP	
S1-1	0.118
S1-2	0.027
S1-3	0.019
Group 2 solvent control (S2)	0.012
Group 3 NP	
S2-1	0.053
S2-2	0.034
S2-3	0.031

NP=Nonylphenol, *O. niloticus*=*Oreochromis niloticus*

## Conclusion

It is concluded that NP is a toxic pollutant and has a profound influence on the biochemical, hormonal, and hematological profiles in addition to histopathological alterations of the liver, kidneys, and testes in Nile tilapia, *O. niloticus*. These toxic effects were relatively repaired once exposure ceased which give hope to maintain fish stock by transferring them from polluted water. However, NP accumulates in muscle tissues which represent edible part from fish during the exposure time and even after exposure has ceased which may have human health hazard. It is evident from the above that water pollution with NP has adverse effects on fish health and reproduction, which is reflected on economic development as well as human health.

## Authors’ Contributions

HTHI and HHHM planned the study design, collected and examined samples and drafted and revised the manuscript. Both authors read and approved the final manuscript.
